# GenECG: a synthetic image-based ECG dataset to augment artificial intelligence-enhanced algorithm development

**DOI:** 10.1136/bmjhci-2024-101335

**Published:** 2025-05-31

**Authors:** Neil Bodagh, Kyaw Soe Tun, Adam Barton, Malihe Javidi, Darwon Rashid, Rachel Burns, Irum Kotadia, Magda Klis, Ali Gharaviri, Vinush Vigneswaran, Steven Niederer, Mark O’Neill, Miguel O Bernabeu, Steven E Williams

**Affiliations:** 1 School of Biomedical Engineering & Imaging Sciences, King’s College London, London, UK; 2 Guy’s and St Thomas’ NHS Foundation Trust, London, UK; 3 Neurolabs, Edinburgh, UK; 4 Centre for Medical Informatics, Usher Institute, University of Edinburgh, Edinburgh, UK; 5 Centre for Cardiovascular Science, University of Edinburgh, Edinburgh, UK

**Keywords:** Artificial intelligence, Data Science

## Abstract

**Objectives:**

An image-based ECG dataset incorporating visual imperfections common to paper-based ECGs, which are typically scanned or photographed into electronic health records, could facilitate clinically useful artificial intelligence (AI)-ECG algorithm development. This study aimed to create a high-fidelity, synthetic image-based ECG dataset.

**Methods:**

ECG images were recreated from the PTB-XL database, a signal-based dataset and image manipulation techniques were applied to mimic imperfections associated with ECGs in real-world settings. Clinical Turing tests were conducted to evaluate the fidelity of the synthetic images, and the performance of current AI-ECG algorithms was assessed using synthetic images containing visual imperfections.

**Results:**

GenECG, an image-based dataset containing 21 799 ECGs with visual imperfections encountered in routine clinical care paired with imperfection-free images, was created. Turing tests confirmed the realism of the images: expert observer accuracy of discrimination between real-world and synthetic ECGs fell from 63.9% (95% CI 58.0% to 69.8%) to 53.3% (95% CI 48.6% to 58.1%) over three rounds of testing, indicating that observers could not distinguish between synthetic and real ECGs. The performance of pre-existing algorithms on synthetic (area under the curve (AUC) 0.592, 95% CI 0.421 to 0.763) and real-world (AUC 0.647, 95% CI 0.520 to 0.774) ECG images containing imperfections was limited. Algorithm fine-tuning with GenECG data improved real-world ECG classification accuracy (AUC 0.821, 95% CI 0.730 to 0.913) demonstrating its potential to augment image-based algorithm development.

**Discussion/conclusion:**

GenECG is the first synthetic image-based ECG dataset to pass a clinical Turing test. The dataset will enable image-based AI-ECG algorithm development, ensuring utility in low resource areas, prehospital settings and hospital environments where signal data are unavailable.

**WHAT IS ALREADY KNOWN ON THIS TOPIC**

- Artificial intelligence-enhanced ECG (AI-ECG) analysis presents a significant opportunity to improve the care of patients with cardiovascular disease.
- Most AI-ECG algorithms have been developed using ECG signal data, limiting their ability to analyse paper-based ECGs which are still prevalent in various hospital and non-hospital settings.

**WHAT THIS STUDY ADDS**

- This study presents GenECG, a high-fidelity, synthetic dataset comprising 21 799 ECG images paired with imperfection-free images and ECG signal data.
- GenECG is the first publicly available synthetic, image-based ECG dataset to pass a clinical Turing test.
- The performance of image-based AI-ECG algorithms improved through fine-tuning with GenECG data, demonstrating the potential for synthetic data to augment AI-ECG research.

**HOW THIS STUDY MIGHT AFFECT RESEARCH, PRACTICE OR POLICY**

- GenECG will facilitate the development of image-based AI-ECG algorithms, promising to expand the application of AI-ECG to traditional hospital settings, reliant on paper-based ECGs and non-hospital environments such as remote healthcare areas or prehospital settings.

## Introduction

 The use of synthetic data in healthcare can facilitate the development of high-fidelity, fully anonymised patient datasets on a previously unachievable scale.^1^ Synthetic patient data can be used as training data to develop artificial intelligence-enhanced (AI) algorithms offering the potential to revolutionise the scope and utility of AI within healthcare settings. Several studies have already highlighted the potential benefits that AI algorithms may offer when applied to the ECG.^2^ For example, AI-ECG can detect electrolyte imbalances,^3^ identify left ventricular systolic dysfunction^4^ and predict risks of paroxysmal arrhythmia^5^ and all-cause mortality.^6^ The ability of AI-ECG to facilitate automated ECG interpretation and detect patterns imperceptible to human observers^2^ presents a significant opportunity to improve the care of patients with cardiovascular disease.

However, despite the potential uses for AI-ECG, current algorithms are primarily limited to analyses of digitised ECG signals rather than ECG images. This is reflective of the composition of currently available public ECG datasets. While multiple signal-based datasets exist,^7^,^9^ the availability of image-based ECG data is limited. To our knowledge, only one publicly available image-based ECG dataset exists.^10^ However, this dataset lacks the aberrations that occur when paper-based ECGs are photographed, referred to hereafter as imperfections, and is substantially smaller than most digital ECG datasets. Nevertheless, numerous healthcare environments continue to rely on printed or scanned ECG images.^11 12^ Given the ongoing, widespread use of paper-based ECGs, a disconnect, therefore, exists between commonly available data types and the AI algorithms designed for their analysis. The creation of a dataset comprising ECG images could enable the development of image-based AI algorithms for use in scenarios where ECG signal data are unavailable. Such a dataset should capture the full range of diversity in paper-based ECGs incorporating visual imperfections common to clinical practice.

The aims of this study were to (1) create a publicly available image-based ECG dataset of labelled images containing visual imperfections typically encountered in routine clinical care; (2) demonstrate the fidelity of the synthetic ECGs by evaluating healthcare professionals’ ability to distinguish them from real-world images and performing a quantitative similarity analysis of synthetic and real-world ECG images and (3) assess whether the performance of pre-existing image-based AI algorithms could be improved through fine-tuning on synthetic data.

## Methods

### PTB-XL dataset

Input ECG signals were provided by the PTB-XL database which contains signal data representing 21 799 clinical ECGs from 18 869 patients.^7^ PTB-XL ECGs are configured as 12 channel binary files with a resolution of 1μv/LSB at 500 Hz (each sample is 0.002 s). Annotated by two cardiologists, there are 71 different ECG statements within the dataset. The statements cover form, rhythm and diagnostic labels in a machine-readable form. The diagnostic labels are organised into 5 superclasses and 24 subclasses as described in Wagner *et al*^7^ (online supplemental table S1 and online supplemental figure S1).

### ECG image generation

For each PTB-XL ECG, an image was created according to recommendations outlined in the ‘AHA/ACCF/HRS Recommendations for the Standardisation and Interpretation of the ECG’ document,^13^ comprising a continuous ten second recording divided into three rows and four columns consisting of 2.5 s of data for each lead where column 1 represents leads I, II and II; column 2 represents aVR, aVL and aVF; column 3 represents V1, V2 and V3 and column 4 represents V4, V5 and V6. An additional rhythm strip containing 10 s of data (lead II) was included for rhythm analysis.

The Blender (Blender Foundation, Amsterdam, Netherlands) software platform was used to create synthetic ECG images using custom code (developed by AB). ECG images were recreated by sampling 2.5 s epochs for each lead, positioned according to AHA/ACCF/HRS recommendations^13^ and with lead markers, lead labels and calibration scales added. Resulting waveform traces were superimposed onto a paper grid (with a resolution set to 150 Hz (25 mm/s) horizontally and 10.0 mm/mV vertically) leading to the generation of a single layout for each PTB-XL ECG. The resolution of the waveform image was set to 5 pixels/mm with a final image output size of 1397×1029 pixels for a 10 s trace.

To validate the accuracy of initial ECG images created from signal data, ECG files representing sine waves of known amplitude (0.5 mV) and frequency (1.25 Hz) were created using the WFDB Toolbox for MATLAB/Octave.^14^ A total of 12 test ECGs were created, consisting of ECGs with the sine wave at a single ECG lead location with all other leads set to a constant electrical potential of 0 mV. These files were converted into ECG images using the same code used for ECG recreation. All validation ECGs were inspected to confirm the correct location of the ECG leads. For each lead of each ECG containing a sine wave, the amplitude and cycle length (frequency) were measured by an observer (NB) blinded to the original amplitude and frequency. Spearman’s correlation coefficient was used to examine the correlation between measured and actual sine wave frequency and amplitude.

### Creation of synthetic ECG images

To apply degradation techniques to ECG images (ie, to make it appear as though images had been photographed), ECGs were passed to a second render which placed each image trace on a 3D model comprising a paper sheet positioned in a synthetically developed workspace. In total, 352 unique geometric variations were created from 8 paper sheet variations, 11 workspaces and 4 synthetic workspace orientations. The Blender platform’s bpy module was used to create an automated Python script for ECG image generation. For each ECG, a mesh and synthetic workspace were randomly selected, and the location and rotation of the ECG paper sheet, camera and light sources were randomly adjusted. To mimic the imperfections associated with photographed ECGs, varying degrees of stucci noise were applied.^15^ This technique, which simulates the appearance of stucco (a wall structure containing holes and bumps), was chosen following a review of the imperfections encountered with real-world ECG photographs by a senior 3D technical artist (AB). For each image, the size and turbulence of the noise were randomly selected to introduce varying degrees of texture distortion.

### Clinical Turing tests

A series of visual Turing tests were designed and conducted to assess the fidelity of synthetic ECG images via an online survey (Qualtrics, Provo, Utah, USA). In all rounds of Turing tests, healthcare professionals were provided with a series of 60 images comprising 30 synthetically created ECGs and 30 photographs of real-world ECGs. ECG images were redacted in areas where text may appear. Images were displayed one-by-one to participants and shown in uniform order. Participants were asked to select whether they thought the images were real or synthetic and, in the second and third rounds, to rate their confidence using a 5-point Likert scale (online supplemental figure S2). At the end of each survey, healthcare professionals were asked to provide qualitative feedback through a series of open questions. Feedback was summarised and used to iteratively improve the dataset’s fidelity. All readers decided whether each image was real or synthetic without any time limit and no prior knowledge regarding the number of real or synthetic images. To avoid bias, healthcare professionals were only allowed to complete one round of clinical Turing tests.

For each round of Turing tests, we measured the accuracy (overall proportion of ECGs correctly identified as ‘real-world’ or ‘synthetic’), true recognition rate (proportion of real-world ECGs identified correctly) and false recognition rate (proportion of synthetic ECGs identified correctly) using adapted terminology from previous Turing tests.^16 17^ The Fleiss-Kappa score was calculated to evaluate the degree of interobserver agreement. For the second and third rounds of clinical Turing tests, confidence Likert scale scores were converted to a signed ordinal scale for area under the curve-receiver operating characteristic (AUC-ROC) score analysis. The data were analysed using SPSS V.29 (IBM).

### Quantitative similarity analysis of real-world and synthetic ECG images

The Fréchet Inception Distance,^18^ a metric which quantifies the similarity between real and synthetic images by comparing the statistical distributions of deep feature representations extracted from a pretrained InceptionV3 neural network, was used to assess the similarity between synthetic and real-world ECG images. The 30 real-world ECGs used for the final round of Turing tests were compared with two sets of synthetic ECG images derived from 30 PTB-XL files: (1) the 30 ECG images from the final round of Turing tests that contained visual imperfections and (2) the 30 corresponding synthetic ECG images without visual imperfections. Fréchet Inception Distance scores were calculated for both sets of images, with lower scores indicating greater similarity to the real-world ECG images. An unpaired t-test was performed to assess the difference in Fréchet Inception Distance scores.

### Assessment of pre-existing image-based algorithms

To examine the performance of currently available image-based algorithms on the GenECG dataset, synthetic images were inputted into two image-based AI-ECG algorithms.^11 19^

The first image-based algorithm tested was ECG-Dx (https://www.cards-lab.org/ecgdx), an automated diagnostic algorithm capable of detecting six diagnoses (atrial fibrillation, sinus tachycardia, sinus bradycardia, left bundle branch block, right bundle branch block and first-degree atrioventricular block). We searched the PTB-XL database for these diagnoses and randomly selected 75 abnormal ECGs. Images with and without image degradation techniques were inputted into the web-based platform. The corresponding classifiers were compared with labels assigned from the PTB-XL dataset.

The second image-based algorithm examined was developed by Bridge *et al,* to distinguish ‘normal’ from ‘abnormal’ ECGs, and this algorithm has demonstrated good performance on scanned ECG printouts.^19^ This model was originally developed using 1172 ECGs and built on InceptionV3,^20^ a pretrained convolutional neural network, with extra layers added to improve performance and prevent overfitting. Due to the unavailability of the original model weights and ECG dataset, we trained an identical model using 1682 images from the ‘ECG images dataset of Cardiac and COVID-19 patients,’ an open-access dataset.^10^ The dataset contains five distinct categories: COVID-19 (n=250), myocardial infarction (n=74), abnormal heart beat (n=546), history of myocardial infarction (n=203) and normal person ECG images (n=859). Given the anticipated challenges in distinguishing normal versus abnormal ECGs in COVID-19 patients, we excluded images from the COVID-19 category. The remaining 1682 images were defined as normal (n=859) or abnormal (n=823), and randomly split into train (n=1082), validation (n=200) and test datasets (n=400).

To assess the algorithm’s ability to analyse synthetic data, we searched the PTB-XL dataset for ECGs which the algorithm would class as either ‘normal’ or ‘abnormal’ and randomly selected 215 GenECG images (96 normal, 119 abnormal) containing image degradation techniques. The images were randomly split into train (n=150), validation (n=22) and test (n=43) images. The trained model was applied to evaluate its performance on the 43 test synthetic ECG images. Following the initial results, the model had low efficiency on synthetic GenECG images since it was not exposed to images that resembled our images during training. Therefore, the model was fine-tuned on the train and validation images (n=172) using weights from the model trained on the open-access dataset as initial weights. The additional layers of the Bridge *et al* model were adjusted accordingly.

To assess the generalisation power of the synthetic model and to ensure that the model did not over-fit during fine-tuning, we re-evaluated the trained synthetic model over the 400 test dataset images derived from the ‘ECG images dataset of Cardiac and COVID-19 patients’ dataset.^10^ Additionally, we evaluated the performance of both the initially developed model and the fine-tuned model on 79 real-world ECG images obtained using methodology described by Sangha *et al*.^11^ Images which would have been defined as either normal (n=24) or abnormal by the Bridge *et al,* algorithm (five abnormal ECGs for each label of interest: sinus arrhythmia, atrial fibrillation, atrial flutter, premature atrial contraction, premature ventricular contraction, atrioventricular block, ventricular tachycardia, supraventricular tachycardia, Wolff-Parkinson-White syndrome, paced rhythm, junctional rhythm)^19^ were obtained from both the life in the fast lane website (https://litfl.com/ecg-library/) and Google searches. The images contained visual imperfections typically encountered in routine clinical care.

ECG images were preprocessed by cropping the region of interest using the rembg Python library (https://pypi.org/project/rembg/2.0.28/) to remove the background of each image. Images were then resized using the Lanczos method to ensure uniform input into the image-based model.^21^ Subsequently, AUC-ROC analysis was performed to evaluate model performance.

## Results

### ECG recreation

**Figure 1:**
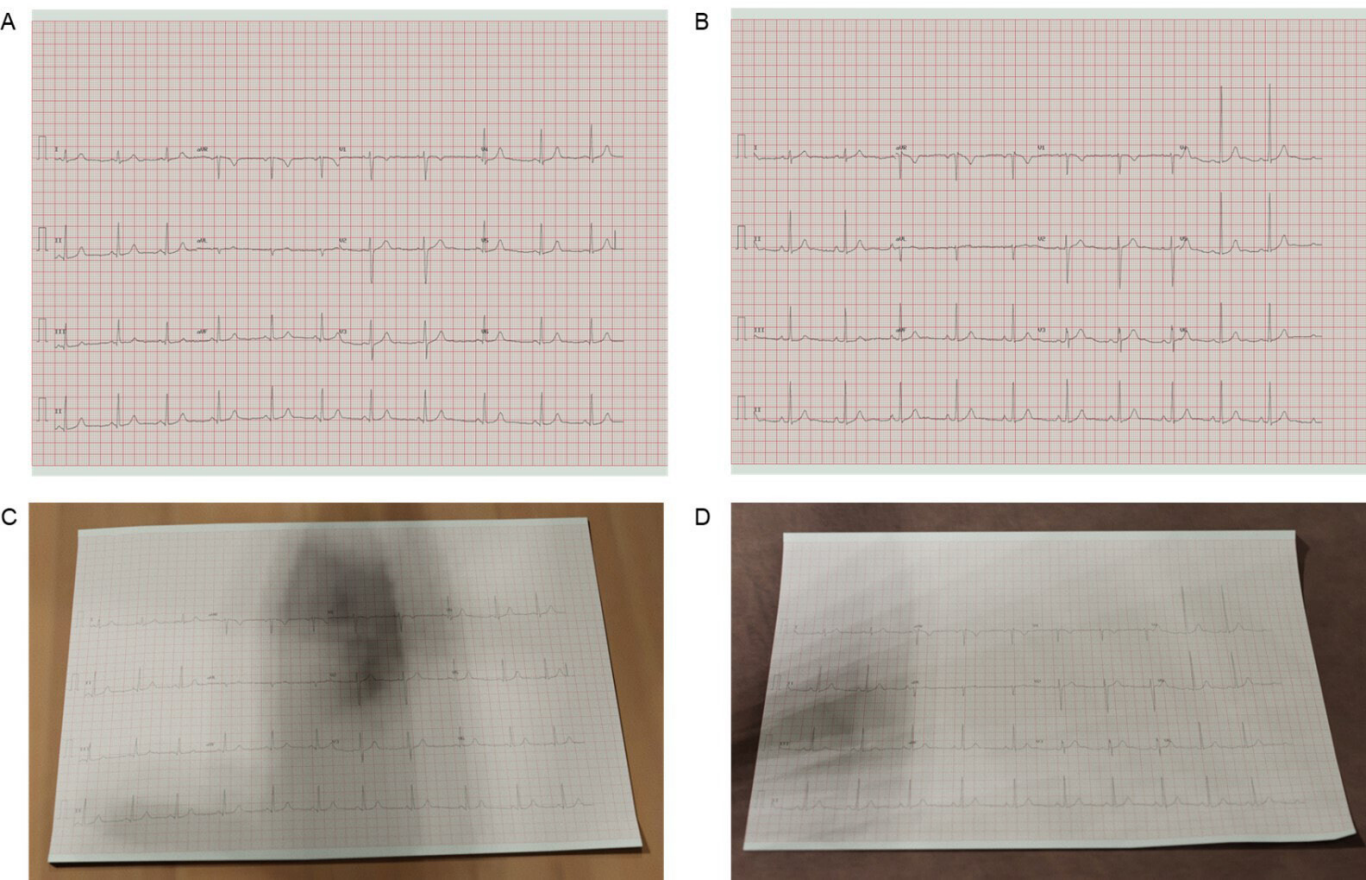
Synthetic ECG images recreated from the PTB-XL dataset. (A, B) Imperfection-free images. (C, D) The same images following the application of image degradation techniques to make it appear as though the images have been photographed. A, C have been recreated from 00074_hr_1R.dat. B, D have been recreated from 00067_hr_1R.dat.

Synthetic ECG images were created for all 21,799 PTB-XL ECGs (dataset A: ECGs without imperfections) (figure 1A,B). Technical validation confirmed that all ECG leads were plotted in the correct location. There was a perfect correlation between measured and actual sine wave amplitude (R=1.0, p<0.001) and frequency (R=1.0, p<0.001) for each ECG lead.

### Clinical validation

**Figure 2:**
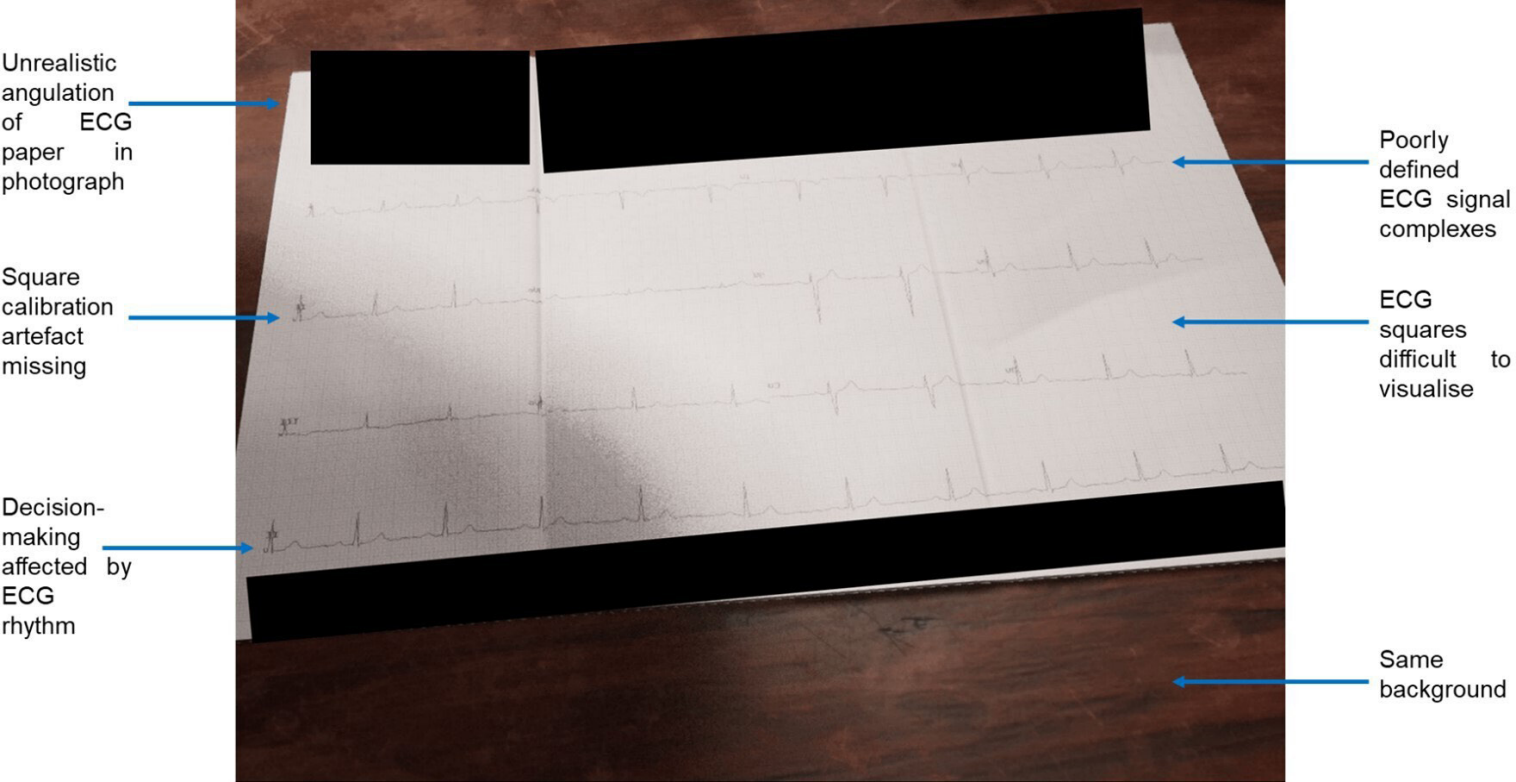
Example of a synthetic ECG used in the initial Turing test with a summary of the qualitative feedback provided by healthcare professionals.

**Figure 3:**
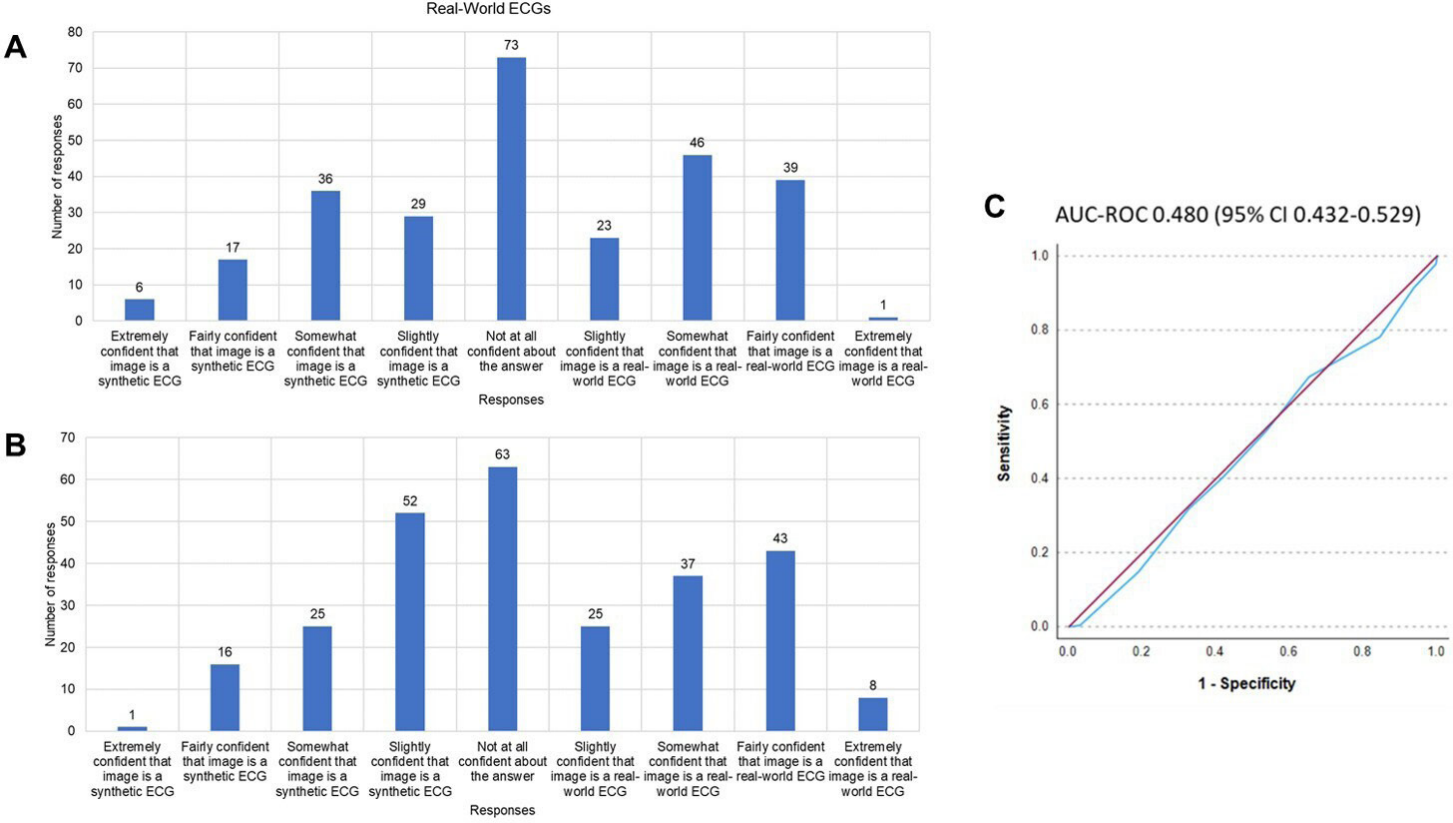
Confidence levels in ECG classification for Turing test round 3. (**A, B**) The number of responses for each option for (**A**) real-world ECGs and (**B**) synthetically created ECGs. (C) An area under the curve-receiver operating characteristic (AUC-ROC) curve examining the impact of confidence level on ability to correctly identify an ECG image as ‘real-world’ or ‘synthetically created.’

Image degradation techniques were applied to a randomly selected subset of synthetic ECGs for clinical validation. The results of the initial two rounds of Turing tests indicated that healthcare professionals were able to distinguish real-world ECGs from synthetic images (round 1 accuracy 63.9% (95% CI 58.0% to 69.8%), round 2 accuracy 59.8% (95% CI 55.9% to 63.7%)) (online supplemental table S2). Qualitative feedback (summarised in figure 2) was used to iteratively improve the fidelity of the ECG images. In the third round of Turing tests, the accuracy, true recognition rate and false recognition rate were 53.3% (95% CI 48.6% to 58.1%), 53.0% (95% CI 48.7% to 57.2%) and 53.7% (95% CI 47.4% to 60.0%), respectively (online supplemental table S2). The Fleiss-Kappa score of 0.049 (95% CI 0.007 to 0.092) indicated a high degree of interobserver variability. The AUC-ROC curve score was 0.480 (95% CI 0.432 to 0.529) indicating that level of confidence was not an accurate predictor of an observer’s ability to distinguish real-world ECG from synthetic images (figure 3).

On completion of the ‘Turing tests’, the image degradation algorithm was deemed capable of recreating life-like ECGs from the PTB-XL database. A dataset of 21 799 images containing PTB-XL ECGs with the incorporation of visual imperfections common to photographed images was created (dataset B: ECGs with imperfections) (figure 1C,D; online supplemental figure S3).

### Quantitative similarity analysis

The mean Fréchet Inception Distance score for the synthetic ECG images containing visual imperfections was 222.23±9.67, while the mean Fréchet Inception Distance score for the imperfection-free synthetic ECG images was 346.98±10.30 (p<0.05) indicating that the imperfection-containing synthetic ECG images more closely resembled real-world ECG images than their imperfection-free counterparts.

### Assessment on pre-existing image-based algorithms

**Figure 4:**
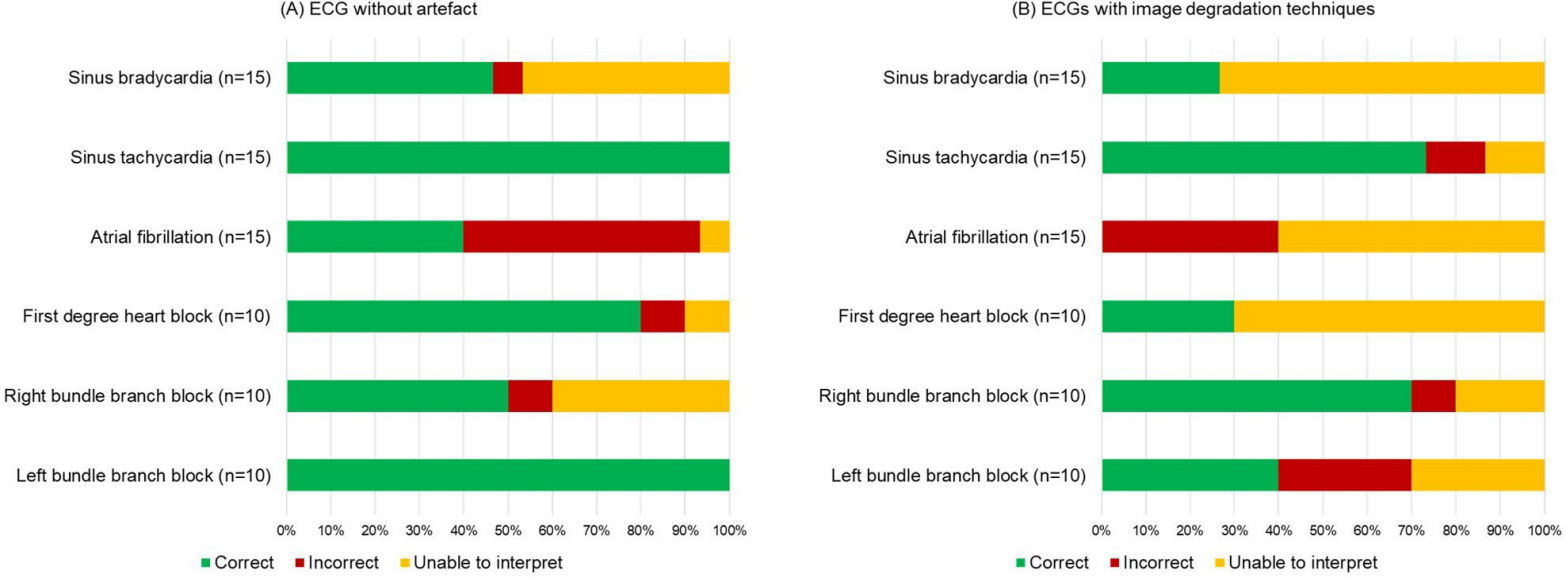
Performance of ECG-Dx image-based algorithm on abnormal images obtained from the PTB-XL dataset. Diagnoses along the y-axis represent PTB-XL labelled diagnoses. The label ‘unable to interpret’ corresponds to an output of ‘none’ from the image-based algorithm.

We assessed the performance of 75 abnormal ECGs both with and without visual imperfections on the ECG-Dx image-based algorithm.^11^ The algorithm was able to correctly identify abnormal diagnoses for 51/75 (68%) imperfection-free images but only 29/75 (39%) images containing imperfections (figure 4).

**Figure 5:**
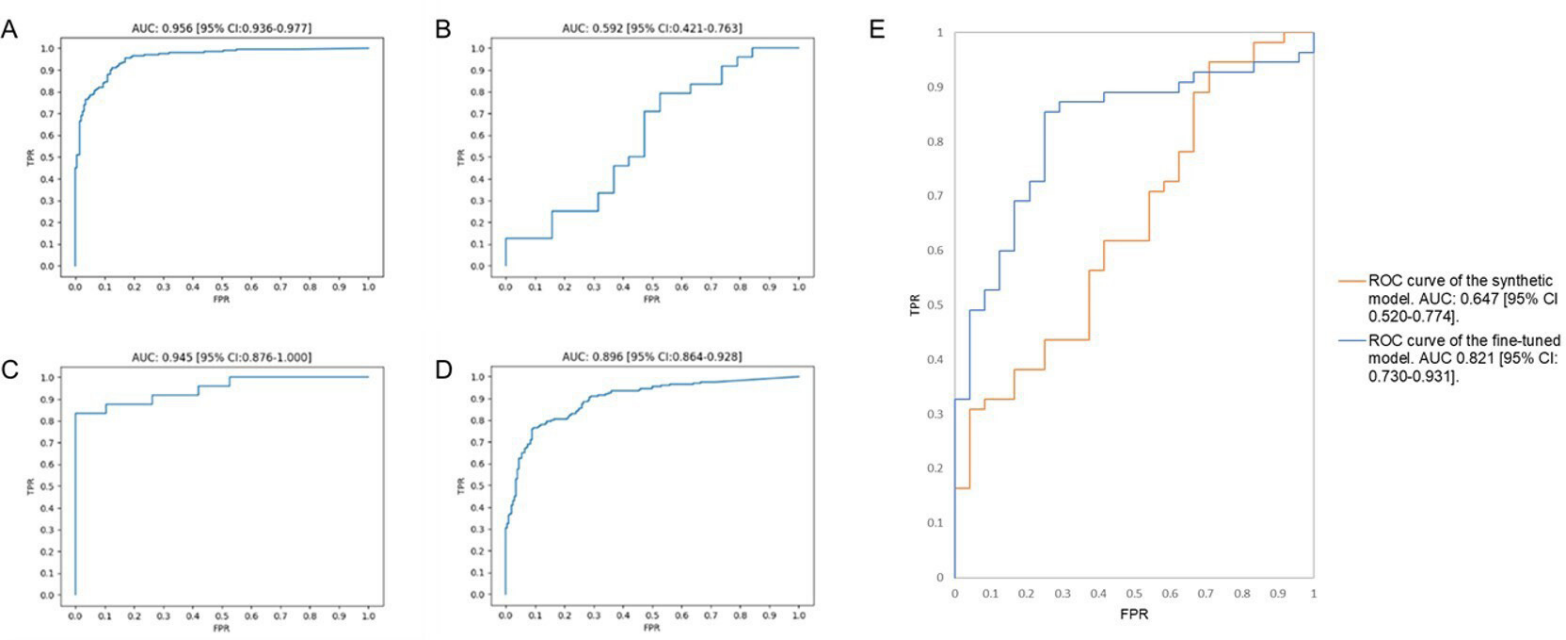
Area under the curve-receiver operating characteristic score curves for ECG images on the algorithm initially developed by Bridge *et al*.^19^ (**A**) The performance of the model on 400 test images obtained from a real-world image-based dataset,^10^ (**B**) the performance of the trained model on 43 test synthetic ECG images containing visual imperfections, (**C**) the performance of the trained model on the same synthetic ECG images following fine-tuning, (**D**) the performance of the fine-tuned model on the 400 test images from the real-world image-based. dataset, (**E**) the performance of both the initially developed synthetic model (orange) and fine-tuned model (blue) on 79 real-world ECG images containing visual imperfections. AUC, area under the curve; FPR, false positive rate; ROC, receiver operating characteristic; TPR, true positive rate.

Using the same architecture as an image-based model developed by Bridge *et al,*^19^ we trained a synthetic model with imperfection-free images from an open access real-world dataset. The synthetic model initially achieved an AUC score of 0.956 (95% CI 0.936 to 0.977) on 400 test dataset images (figure 5A). The model was less able to distinguish normal from abnormal on 43 GenECG images (AUC score 0.592, 95% CI 0.421 to 0.763) (figure 5B). Fine-tuning of the model led to a substantial improvement in image classification on the same 43 images (AUC score 0.945, 95% CI 0.876 to 1.000) (figure 5C). The fine-tuned model subsequently achieved an AUC score of 0.896 (95% CI 0.864 to 0.928) on 400 real-world ECGs demonstrating that the model did not over-fit during fine-tuning (figure 5D). In addition, we assessed the performance of both the initially developed synthetic and the fine-tuned model on 79 real-world ECG images containing imperfections (figure 5E). The initially developed synthetic model achieved an AUC of 0.647 (95% CI 0.520 to 0.774) while the fine-tuned model achieved an AUC of 0.821 (95% CI 0.730 to 0.913) demonstrating an improvement in performance following model fine-tuning on GenECG data.

## Discussion

In the present study, we used a publicly available signal-based dataset to create GenECG—a high-fidelity, synthetic image-based ECG dataset comprising 21 799 ECGs with imperfections encountered in routine clinical care paired with imperfection-free images. Clinical Turing tests confirmed the ECGs were indistinguishable from real-world ECGs. Quantitative similarity analysis demonstrated that imperfection-containing ECG images more closely resembled real-world ECGs than their imperfection-free counterparts. Pre-existing image-based AI algorithms exhibited good performance levels on synthetic images without imperfections, but poor performance on synthetic images with imperfections. Importantly, existing algorithm accuracy on real-world ECG images could be improved following fine-tuning with GenECG data. These findings highlight the potential for synthetic ECG data to augment clinically useful image-based AI-ECG algorithm development.

Signal-based AI-ECG algorithms have previously demonstrated diagnostic capabilities comparable to experienced clinicians^22 23^ and the ability to detect subtle ECG patterns, facilitating both improved screening and phenotyping of disease.^24^ However, the requirement for signal data presents a barrier in many clinical areas where signal data are unavailable.^12^ Such areas include low resource areas, prehospital settings and hospital environments where paper-based ECGs continue to be used. To address this issue, digitisation tools have been developed to derive signal data from paper-based ECGs.^25^,^27^ Unfortunately, external validation of these tools is limited by a relative unavailability of image-based data, making it difficult to assess digitisation methods.^28^ GenECG provides a benchmark dataset which could be used with PTB-XL signal data^7^ to enable external validation of ECG digitisation tools. Alternatively, the development of image-based algorithms using GenECG could obviate the requirement to digitise paper-based ECGs altogether. Given the absence of a universal format for digitised ECG data storage and exchange,^29^ this option would offer additional clinical utility.

In the present study, we used an existing signal-based dataset to create a large-scale image-based ECG dataset. In creating the images from the PTB-XL database, GenECG benefits from the PTB-XL dataset’s broad scope and variety.^7^ Moreover, we have demonstrated the incorporation of imperfections into GenECG, thereby ensuring that the dataset represents a real-world clinical dataset as closely as possible. The quantitative similarity analysis and clinical Turing tests confirmed that our dataset is life-like, with generated images incorporating the imperfections typically encountered with real-world ECGs. To our knowledge, GenECG is the first synthetic image-based ECG dataset to pass a Turing test.

The performance of pre-existing image-based algorithms on both real-world ECGs and our GenECG data indicates poor generalisability of existing algorithms to real-world settings. In training and fine-tuning the model developed by Bridge *et al*,^19^ we have demonstrated that using synthetic ECG data may overcome the limited efficacy of pre-existing image-based algorithms on real-world ECG images. Importantly, the performance of the fine-tuned model on real-world images has also demonstrated the potential for synthetic data to be used to improve the performance of image-based AI-ECG algorithms. Our synthetic ECG images provide a large data repository which can be used to facilitate the development of AI-ECG algorithms on images containing image degradation techniques. This will ensure the translation of AI-ECG analysis from the research setting to the clinical workplace.

### Limitations

In this study, ECGs were reconstructed from clinically recorded signal data. While the Turing tests confirmed the fidelity of the images created, whether fully synthetic ECG images can be created is not yet known. Moreover, this study did not specifically evaluate the impact of individual visual imperfections, such as filtering interference or noise, on the performance of image-based AI-ECG algorithms. Future research could expand on our study by introducing more image degradation techniques and examining how specific sources of degradation, such as paper angulation, distortion and noise, affect algorithm accuracy.

A further limitation is that the ECGs created are of a single layout type. While several major cardiac societies have advocated a standardised simultaneous lead format for paper-based ECGs,^13^ ECG layouts can vary in clinical practice. It is, therefore, crucial to ensure that future image-based datasets capture this variability such that image-based AI algorithms can be applied to ECGs regardless of the ECG layout encountered.

An additional limitation of the study was that only two classification algorithms were studied, ECG-Dx which can detect six rhythm disturbances,^11^ and the algorithm developed by Bridge *et al,* which classifies ECGs as either ‘normal’ or ‘abnormal.’^19^ To better understand how GenECG can augment image-based AI-ECG algorithm development, it is essential to develop and assess a broader range of algorithms. A significant step in this direction was the recent open challenge hosted by the British Heart Foundation Data Science Centre,^30^ which encouraged multiple research groups to create image-based AI-ECG algorithms using GenECG. The public availability of GenECG will provide researchers with a resource to facilitate image-based AI-ECG research. Future efforts should focus on validating algorithms developed with GenECG across various medical institutions and patient populations to ensure their generalisability and clinical utility. Such validation studies are an integral step towards the eventual integration of image-based AI-ECG algorithms into clinical workflows.

## Conclusions

This study presents GenECG—a high-fidelity, synthetic image-based ECG dataset comprising 21 799 ECG images paired with imperfection-free images and ECG signal data. Clinical Turing tests confirmed the fidelity of the synthetic images, demonstrating their potential to facilitate image-based AI ECG algorithm development. Our findings demonstrate the ability of synthetic data to enhance the performance of pre-existing image-based algorithms. GenECG will enable the development of image-based AI-ECG algorithms promising to bridge the gap between AI-ECG research and clinical practice. This will ensure that AI-ECG can be used in hospital settings, where paper-based ECGs continue to be used, and non-hospital settings including remote healthcare areas and pre-hospital settings.

## Supplementary material

10.1136/bmjhci-2024-101335 online supplemental file 1

## Data Availability

Data are available in a public, open access repository.
